# Cross-sectional study for assessment of knowledge, attitudes and practices of chronic kidney disease patients toward potassium-rich diet intake in Jazan-Saudi Arabia

**DOI:** 10.1097/MD.0000000000042260

**Published:** 2025-05-09

**Authors:** Mohammed Somaili, Atheer Akoor, Eman Refaei, Mohsen Deibaji, Abdulrahman Abdullah Aqeel, Saleh Ghulaysi, Areej Siddiq Areeshi, Raghad Abdu Mobaraki, Entsar Ahmed Qadah, Naif Gharwi, Abdulla Madkali, Khalid Ahmed Muafa, Abdulrahman Hakami, Mona Alshahrani

**Affiliations:** a Internal Medicine Department, Faculty of Medicine, Jazan University, Jazan, Saudi Arabia; b Internal Medicine Department, Faculty of Medicine, King Khaled University, Jazan, Saudi Arabia.

**Keywords:** attitudes, chronic kidney disease, jazan, knowledge, perception, potassium intake, Saudi Arabia

## Abstract

Hyperkalemia is prevalent among chronic kidney disease patients, and their knowledge and perceptions regarding a potassium-rich diet vary widely. In this study, we aim to evaluate the level of knowledge regarding potassium-rich diet Intake among chronic kidney disease patients in Jazan, Saudi Arabia, and to identify potassium-rich diet intake-related practices among them. We conducted a cross-sectional questionnaire-based study using the convenient sampling method on CKD patients. The questionnaire was composed of 20 questions distributed over 3 domains. The data had been analyzed using SPSS software version 23. Frequency and percentages were used to display categorical variables. Mean and standard deviation was used to present numerical variables. The independent t-test and analysis of variance (ANOVA) test were both used to test for factors associated with knowledge and perception score. A total of 404 were involved in the study. Most were aged between 41 and 60 years 38.6%. The majority were male 57.2% and of Saudi nationality 76.7%. Regarding marital status, 64.6% were married and 4% were divorced. Regarding employment, 67.3% were unemployed, and 5% were students. As regards education, 60.9% had less than a high school education and 0.9% had a master’s degree/PhD or equivalent. The knowledge levels about potassium-containing diets show that 79.8% had low knowledge, 20% had moderate knowledge, and only 0.2% had a high knowledge level. Participants’ behaviors toward a potassium-rich diet show that 52.7% have considered lowering dietary potassium is essential, while 32.7% were unsure of its necessity. Only 16.1% indicated that salt quantity on nutritional labels influenced their shopping choices. A total of 53% of participants avoided or reduced consumption of potassium-rich foods. Additionally, 36.4% opted for low-potassium alternatives and 26% read potassium content on labels. Behavioral scores ranged from 0 to 5, with a mean of 1.84 ± 1.62. Lastly, A moderate positive correlation was found between knowledge and behavior scores. This study identifies significant gaps in knowledge and behavior regarding high-potassium meals among CKD patients in Jazan, Saudi Arabia. The results underscore the necessity for better patient education, customized treatment plans, and a multidisciplinary approach to nutrition management to enhance dietary adherence and clinical outcomes.

## 1. Introduction

The incidence and prevalence of chronic kidney disease are rising globally, making it a public health concern.^[1,2]^ It is estimated that there are 850 million individuals with chronic kidney disease (CKD) worldwide^.[3]^.Reduced glomerular filtration rate (GFR) to less than 60 mL/min per 173 m^2^, increased urine protein excretion to more than 30 mg/g, or both, for a minimum of 3 months, are indicators of CKD.^[4,5]^ Due to decreased renal excretion, patients with CKD may experience hyperkalemia, hyperphosphatemia, anorexia, and muscle and fat loss. There are 5 stages of CKD, and the danger of these chemicals building up increases with the advanced stages of CKD.^[6]^

Patients with chronic kidney disease usually have a higher prevalence rate of hyperkalemia compared to the general population due to defective excretion.^[7]^ More advanced stages of CKD are associated with a higher prevalence rate of hyperkalemia. In the early stages, it makes up 14% to 20%, whereas in advanced CKD without dialysis, it makes up 25% to 40%.^[8,9]^ Reduced urine excretion, the use of high blood pressure medication like angiotensin-converting enzyme inhibitors or increased intake of potassium-rich diets are linked to the development of hyperkalemia in patients with CKD.

CKD patients’ ignorance of a diet high in potassium may increase their chance of developing hyperkalemia.

In 2011, a meta-analysis of eleven studies totaling 247,510 people evaluated the association between regular potassium intake and the development of cardiovascular disease. They found that higher dietary potassium consumption has been linked to a decreased risk of stroke and total cardiovascular disease. These findings back up the advice to eat more potassium-rich foods to prevent vascular disease.^[10]^ However, the case is different in CKD patients due to the reasons mentioned above. A 2-year study on 678 CKD patients (pre-dialysis and dialysis patients) was undertaken in Bucharest, Romania in 2011. The goal of the study was to examine how much serum potassium changes are associated with the development of arrhythmias in CKD patients. They looked at all acute events that caused hospitalization in pre-dialysis patients. The authors of this study concluded that even though hypokalemia is a more significant risk factor than hyperkalemia in triggering arrhythmias, even little variations in blood potassium levels might also cause arrhythmia and increase mortality rates in CKD patients.^[11]^

In 2021, 266 individuals with non-dialysis dependent chronic kidney disease (NDD-CKD) and a glomerular filtration rate (GFR) of less than 45 mL/min from an urban nephrology clinic in the United States completed a questionnaire to assess their awareness of diet restriction and salt food sources. The findings of this study revealed that hyperkalemic patients were more aware of the potassium diet restriction than non-hyperkalemic patients. However, more than 50% of study participants were unaware of potassium sources in their diets.^[12]^

To our knowledge, no prior research has evaluated the knowledge and perception of potassium-rich diet intake among patients with chronic renal disease in Saudi Arabia, so this study aims to address this topic and provide an overview of the current situation in this regard.

## 2. Research methodology

### 2.1. Study design

This study was designed to be an observational cross-sectional during a period from June 2023 till the end of December 2023.

### 2.2. Study setting

The study has been conducted in the Jazan region. Jazan region is located in the Southern part of Saudi Arabia and it includes 13 governorates with more than 1.500.000 people distributed in different governorates, towns, and villages. The study was conducted in renal clinics at Prince Mohammed Bin Naser Hospital (PMBNH) and Alomais Medical Center (AMS). PMBN Hospital is a governmental facility which inaugurated in November 2015. It is located in Jizan city and has a 200-bed capacity. AMS is a private hospital with a 150-bed capacity. The 2 hospitals serve around 160 CKD patients per month (2000 patients a year). The counseling and workup of renal replacement therapy for most advanced CKD patients are also performed in these hospitals and then referred to tertiary hospitals in big cities of the kingdom for the institution of renal replacement therapy.

### 2.3. Study population

Participants who fulfill the inclusion criteria had been involved in this study:

### 2.4. Inclusion criteria

All mentally competent adult individuals (>18 years) who have established diagnosis with stage 3 to 5 CKD, regardless of the cause of CKD and visiting the clinics as new or follow-up, have been enrolled in this study. The staging is based on the Kidney Disease Initiative Global Outcome (KDIGO) classification of estimated glomerular filtration rate in CKD patients, which ranges from 30 to 60 mL/min/1732 (stage 3) down to < 15 ml/min/1732 (stage 5) for 3 months or more.^[13]^

### 2.5. Exclusion criteria

End-stage renal disease on any modality of renal replacement therapy including: peritoneal dialysis, or kidney transplant patients were excluded from this study. Pediatric population, adults who refuse to participate or people with any psychiatric illnesses or lack of judgment ability have been excluded as well.

### 2.6. Data collection tool

A pretested self-administered questionnaire has been adopted from various previous studies and translated into the Arabic language. As there is no questionnaire specific to potassium diet in CKD patients, the chronic kidney disease short food frequency questionnaire has been modified to be the data collection tool for this study.^[14]^ The questionnaire items had been modified to fit the research objectives and to be aligned with the usual Arabic foodstuff that is enriched with potassium. A pilot study on 10 to 15 patients was performed to avoid bias or any technical issues. Any ambiguities in the questions or responses were removed before their implementation. The questionnaire consists of a total of 20 questions distributed over 3 domains. The first section is about demographics and patients’ clinical characteristics and is composed of 10 questions. The second domain is about knowledge assessment of a potassium-rich diet. This section is composed of 5 questions covering the knowledge about the clinical significance of hyperkalemia in patients with CKD as well as the potassium contents in various food kinds of stuff. A total of 44 food types from different food sources was included in this questionnaire after confirmation of alignment with usual Arab food consumption. The type of foodstuff and the diet classification into high or low potassium had been adopted from National Kidney Foundation patient educational materials and the World Health Organization website.^[15,16]^ The last 5 questions are about the behavior of CKD patients toward a potassium-rich diet. For example, the patients’ practices to control the dietary sources of a potassium-rich diet and the potential barriers for doing so. The patient earned one point for each correct answer and “0” for a wrong or “I don’t know” answer. The participant was considered adequately knowledgeable if he/she answered 50% (26 out of 52 questions) or more of the questions answered correctly, and with inadequate knowledge, if the correct answers were less than 50%.

### 2.7. Study sampling

All patients who fulfilled the inclusion criteria with an established diagnosis of stage 3 to 5 CKD visited the renal clinics at PMBNH and AMS in Jazan during the pre-specified duration. So, there was be no specific method of sampling.

### 2.8. Statistical analysis

Data analysis was performed using Statistical Package for the Social Sciences, SPSS 23rd version. Frequency and percentages were used to display categorical variables. Minimum, maximum, mean, and standard deviation were used to display numerical variables. Independent *t* test and ANOVA test were used to test factors associated with knowledge score and with behavior score. ANOVA test was followed by Tukey post hoc test to reveal where the exact difference between groups exist. Pearson’s correlation was used to test for correlation between knowledge score, and behavior score. Level of significance was set at .05.

### 2.9. Ethical consideration

Ethical approval was obtained from Standing Committee for Scientific Research – Jazan University (reference no.: REC-43/10/233). All data was kept private and only accessible when necessary for scientific research purposes.

## 3. Results

### 3.1. Study participants’ baseline characteristics

In total, 404 participants were included in this study. Table 1 shows the participant’s socio-demographic profiles. Of the participants, 30.4% were between 20 and 40 years old, 38.6% were between 41 and 60 years old, 26.5% were between 61 and 80 years old, and 4.5% were older than 80 years old. Fifty-seven percent were male and 43% were female. Regarding nationality, 76.7% were Saudis. In terms of marital status, 64.6% of participants were married, 31.4% were single, and 4% were divorced. Regarding occupational status, 27.7% reported being currently employed, about two-thirds of participants reported that they are unemployed currently, and 5% reported being students. As for monthly income, 64.4% reported having an income of less than 4000 SR, 15.3% reported having an income between 4000 and 7999 SR, 14.9% reported having an income between 8000 and 14,999 SR only 5.4% reported having an income of more than 25,000 SR. Regarding living area status, 71.3% of participants reported living in a rural area, while 28.7% reported that they were living in an urban area. In terms of their education levels, 60.9% of participants had less than a high school education, 14.4% had a high school education, 9.4% had a diploma, 14.4% had a bachelor’s degree, and 0.9% had a master’s degree/PhD or equivalent (Table 1).

**Table 1:** Socio-demographic profile of the participant (n = 404).

Demographical characteristics	n	%
Age		
20–40 years	123	30.40
41–60 years	156	38.60
61–80 years	107	26.50
Older than 80 years	18	4.50
Gender		
Male	231	57.20
Female	173	42.80
Nationality		
Saudi	310	76.70
Non-Saudi	94	23.30
Marital status		
Married	261	64.60
Single	127	31.40
Divorced	16	4.00
Occupation status		
Currently employed	112	27.70
Currently unemployed	272	67.30
Student	20	5.00
Monthly income		
<4000 SR	260	64.40
4000–7999 SR	62	15.30
8000–14999 SR	60	14.90
15,000–24,999 SR	19	4.70
> 25,000 SR	3	0.70
Living area		
Rural	288	71.30
Urban	116	28.70
Education status		
Less than high school	246	60.90
High school	58	14.40
Diploma	38	9.40
Bachelor’s degree	58	14.40
Master’s/PhD or equivalent	4	0.90

In terms of the best-known kidney function, 61.9% reported that they don’t know how severe is their kidney function. Nine percent had stage 3 chronic kidney disease, 7.2% had stage 4 chronic kidney disease and 21.8% had stage 5 chronic kidney disease.

**Figure 1.**
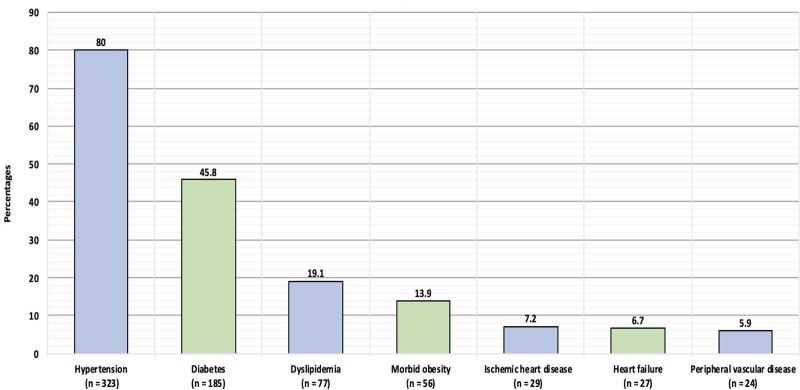
Comorbidities.

Figure 1 shows the comorbidities of the study participants. Of the participants, 80 % had hypertension, 45.8% had diabetes, 19.1% had dyslipidemia, 13.9% had morbid obesity, 7.2% had ischemic heart disease, 6.7% had heart failure, and 5.9% had peripheral vascular disease.

**Figure 2.**
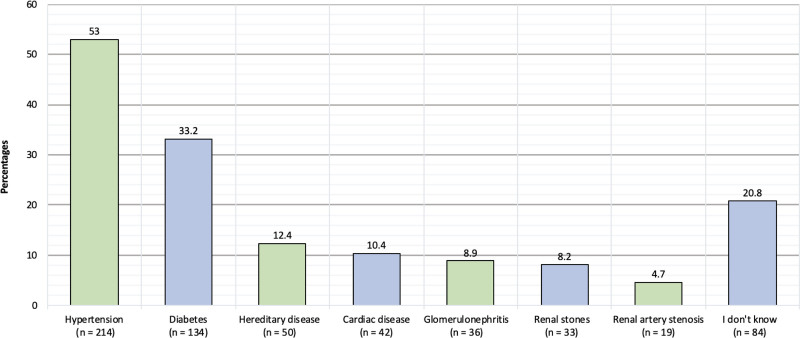
Causes of chronic kidney disease among the participants.

The principal cause of chronic kidney disease is illustrated in Figure 2. The cause of chronic kidney disease for 53% was hypertension; 33.2% was diabetes; 12.4% was a hereditary disease, 10.4% was a cardiac disease, 8.9% was glomerulonephritis, 8.2% was nephrolithiasis, 4.7% was renal artery stenosis, 20.8% of participants reported that they don’t know.

### 3.2. Assessment of participants knowledge towards potassium-containing diets

The participants’ knowledge of potassium-containing diets had been assessed in this study. The participants stated that they had received previous instructions on this topic, and only 55.4% knew about potential health issues linked to excessive potassium consumption. The awareness of the specific health hazards associated with high potassium levels ranged from 28.7% to 38.6% for various symptoms, indicating a moderate level of understanding. A significant number of participants (80.4%) demonstrated a lack of awareness of the recommended maximum daily potassium intake for individuals with CKD. Only 13.1% of the participants accurately identified recommendations for daily potassium consumption. There was considerable variation in the level of awareness regarding the potassium content of the different foods. Familiar foods known to be high in potassium, such as bananas, have a higher level of understanding (54.7% accuracy). In contrast, less obvious options, such as broccoli, had a lower level of awareness (13.4% accuracy). There was a notable level of uncertainty, as evidenced by the considerable proportion of responses that indicated “I don’t know” for all food items (ranging from 36.6% to 85.6%). The knowledge scores, ranging from 0 to 45, had an overall mean of 13.69 out of 52 (SD = 11.3), indicating a low level of knowledge. These findings suggest that patient knowledge regarding potassium-related dietary factors in managing chronic kidney disease is highly variable and inadequate (Appendix Table S1, Supplemental Digital Content, https://links.lww.com/MD/O837).

**Figure 3.**
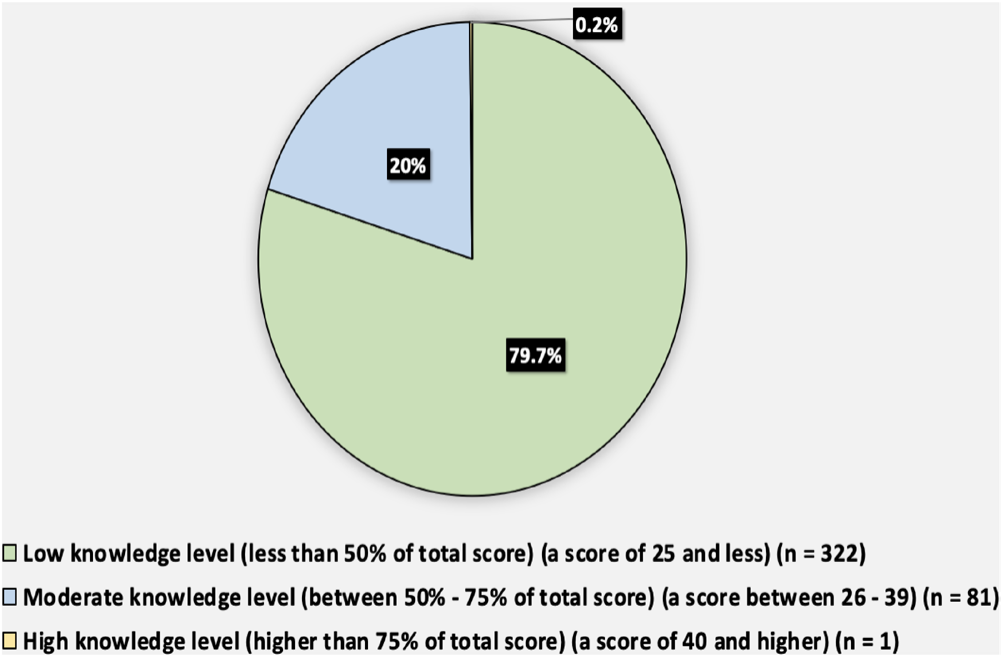
Knowledge level of potassium-containing diet among the participants.

Figure 3 shows the knowledge level of the potassium-containing diet. A total of 79.7% of participants had a low knowledge level (less than 50% of the total score) (a score of 25 or less), 20% had a moderate knowledge level (between 50% and 75% of the total score) (a score between 26 and 39), and only 0.2% had a high knowledge level (higher than 75% of the total score) (a score of 40 and higher).

### 3.3. Assessment of participants behavior and practice towards potassium-containing diets

Behavior and practice towards potassium-containing diets were also assessed in this study. 52.7% of participants reported that lowering the potassium in their diet was essential, while 32.7% did not know if it was necessary. Only 16.1% of participants reported that information about salt quantity on nutritional labels affected their grocery shopping choices. A total of 53% of participants reported that they are avoiding eating potassium-rich products or reducing their consumption. In total, 36.4% reported buying alternative products with a low potassium content, and 26% reported reading the potassium content on the nutritional labels. The minimum behavioral score was 0, the maximum was 5, and the mean was 1.84 ± 1.62 (Appendix Table S2A and S2B, Supplemental Digital Content, https://links.lww.com/MD/O838).

Table 2 presents the participants’ barriers to noncompliance with the K-rich diet. According to the study findings, individuals with chronic kidney disease encounter considerable difficulties when adhering to high-potassium diets. A significant hurdle encountered by most participants (61.1%) was difficulty selecting appropriate meals. Most respondents reported facing significant challenges in managing their diet. Approximately 54.7% of the participants reported difficulty monitoring their nutrient intake, while approximately 47.3% faced difficulty accurately estimating portion sizes. Social and lifestyle factors had a significant impact, as more than half of the participants reported difficulties managing their diet when surrounded by family or friends. Additionally, nearly half of the individuals struggled to maintain dietary control when feeling hungry, and a significant proportion faced challenges when occupied with tasks. Many people have struggled to understand CKD diets and consider it as complicated. Nearly half of the participants (45.8%) found it challenging, whereas a similar percentage (43.1%) felt deprived. Additionally, 43% of participants found that the CKD diet is tasteless or bland. It is worth mentioning that economic factors did not have a significant impact, as only 27.2% of participants considered the diet expensive. Approximately 37.4% of the participants expressed concerns about limited options in food stores, suggesting a moderate issue. It is important to note that most patients understood the benefits of commitment to the CKD diet, with only 22.7% of patients being unable to recognize its advantages. Despite many obstacles, there is a well-established recognition of diet role in managing CKD (Table 2).

**Table 2:** Participants barriers to non-compliance with the potassium-rich diet.

What are the potential barriers to noncompliance with the potassium-rich diet? (more than 1 answer can be chosen)					
Item	Strongly disagree	Disagree	Neutral	Agree	Strongly agree
Difficulty knowing which food to eat	30 (7.4%)	35 (8.7%)	92 (22.8%)	120 (29.7%)	127 (31.4%)
Craving for a potassium-rich diet	40 (9.9%)	48 (11.9%)	142 (35.1%)	104 (25.7%)	70 (17.3%)
CKD diet is bland/tasteless	34 (8.4%)	64 (15.8%)	132 (32.7%)	100 (24.8%)	74 (18.3%)
Difficulty tracking nutrient intake	26 (6.4%)	53 (13.1%)	104 (25.7%)	118 (29.2%)	103 (25.5%)
Feeling deprived with CKD diet	35 (8.7%)	66 (16.3%)	114 (28.2%)	98 (24.3%)	91 (22.5%)
CKD diet looks complicated	36 (8.9%)	62 (15.3%)	121 (30%)	107 (26.5%)	78 (19.3%)
Difficulty controlling diet when hungry	34 (8.4%)	69 (17.1%)	105 (26%)	99 (24.5%)	97 (24%)
Difficulty controlling diet with family/friends	30 (7.4%)	72 (17.8%)	92 (22.8%)	97 (24%)	113 (28%)
Difficulty controlling diet when busy	36 (8.9%)	70 (17.3%)	122 (30.2%)	90 (22.3%)	86 (21.3%)
Difficulty estimating portion sizes	34 (8.4%)	54 (13.4%)	125 (30.9%)	109 (27%)	82 (20.3%)
CKD diet is too expensive	52 (12.9%)	90 (22.3%)	152 (37.6%)	64 (15.8%)	46 (11.4%)
Cannot see the benefits of the CKD diet	85 (21%)	108 (26.7%)	119 (29.5%)	43 (10.6%)	49 (12.1%)
Gregory stores have a limited selection	46 (11.4%)	50 (12.4%)	157 (38.9%)	84 (20.8%)	67 (16.6%)

CKD = chronic kidney disease.

### 3.4. Baseline characteristics associated with knowledge of potassium-containing diets

Table 3 shows the factors associated with knowledge of potassium-containing diets. Age was significantly associated with knowledge scores (*P* < .001), where it was observed that the participants aged 20 to 40 years had the highest knowledge score. Tukey’s post hoc test revealed that those aged 61 to 80 years had significantly lower knowledge scores than those aged 20 to 40 years (*P* < .05) and those aged 41 to 60 years (*P* < .05). Occupation status was also significantly associated with knowledge score (*P* < .001), where it was observed that those who reported being employed had the highest knowledge score. Tukey’s post hoc test revealed that those who reported being employed had a significantly higher knowledge score than those who reported being unemployed (*P* < .05). Monthly income was also significantly associated with knowledge score (*P* = .008), where it was observed that those with a monthly income of 8000 to 14999 SR had a significantly higher knowledge score than those with a monthly income of 4000 to 7999 SR (*P* < .05) and those with monthly income <4000 SR (*P* < .05). Education was also significantly associated with knowledge score (*P* < .001), where it was observed that the higher the education status, the higher the knowledge score, except that those with bachelor’s degrees had higher knowledge scores compared to those with master/PhD or equivalent. Tukey’s post hoc test revealed that those with less than high school education had significantly lower knowledge scores than those with diplomas (*P* < .05) and bachelor’s degrees (*P* < .05). Those with hereditary diseases as the cause of chronic kidney disease had a significantly higher knowledge score than those with non-hereditary disease causes (*P* = .008). A moderate positive correlation was observed between the knowledge and behavior scores (*P* < .001, correlation coefficient = 0.458). Marital status, living area, best-known kidney function, and the following causes of chronic kidney disease (diabetes, hypertension, cardiac disease, glomerulonephritis, renal artery stenosis, and renal stones) were not significantly associated with the knowledge score.

**Table 3:** Factors associated with knowledge toward potassium-containing diet.

Factor	Knowledge score		*P* value
	Mean	Standard deviation	
Age			<.001*
20–40 years	16.96	10.33	
41–60 years	14.22	11.50	
61–80 years	9.5	11.08	
Older than 80 years	11.56	9.61	
Gender			.041*
Male	14.68	11.53	
Female	12.36	10.87	
Marital status			.799
Married	13.73	11.59	
Single	13.39	10.57	
Divorced	15.38	12.51	
Occupation status			<.001*
Currently employed	18.16	11.58	
Currently unemployed	11.65	10.82	
Student	16.25	8.37	
Monthly income			.008*
<4000 SR	12.73	10.94	
4000–7999 SR	12.44	11.23	
8000–14,999 SR	18.30	11.87	
15,000–24,999 SR	15.47	12.07	
>25,000 SR	18.67	6.03	
Living area			.737
Rural	13.81	11.02	
Urban	13.39	12.00	
Education status			<.001*
Less than high school	11.50	11.10	
High school	14.57	10.76	
Diploma	17.82	10.32	
Bachelor’s degree	19.14	10.68	
Master’s/PhD or equivalent	17.25	13.72	
Best known kidney function			.251
Stage 3 chronic kidney disease	17.59	12.86	
Stage 4 chronic kidney disease	18.28	12.35	
Stage 5 chronic kidney disease	14.83	10.36	
Cause of CKD			
Diabetes	13.96	11.14	.729
Hypertension	14.14	10.89	.391
Cardiac disease	15.43	12.41	.291
Hereditary diseases	17.62	10.59	.008*
Glomerulonephritis	15.97	10.78	.204
Renal artery stenosis	18.37	9.77	.064
Renal stones	11.15	10.85	.179
Correlation between knowledge score and behavior score			
*P* value	<.001*		
Correlation coefficient	0.458		

CKD = chronic kidney disease.

* Significant at level .05.

### 3.5. Factors associated with behavior and practice toward potassium-containing diets

Table 4 illustrates the factors associated with the behavior toward a potassium-containing diet. Age was significantly associated with behavior scores (*P* < .001), where it was observed that the participants aged 20 to 40 years had the highest behavior scores. Tukey’s post hoc test revealed that those aged 61 to 80 years had significantly lower behavior scores than those aged 20 to 40 years (*P* < .05) and those aged 41 to 60 years (*P* < .05). Those aged > 80 years also had significantly lower behavioral scores than those aged 20 to 40 years (*P* < .05) and those aged 41 to 60 years (*P* < .05). Males had significantly higher behavior scores than females (*P* = .005) (2.04 ± 1.7 vs 1.58 ± 1.48). Occupation status was also significantly associated with behavior scores (*P* = .003), where it was observed that those who reported being students had the highest knowledge score. Tukey’s post hoc test revealed that those who reported being employed had a significantly higher behavior score than those who reported being unemployed (*P* < .05). Monthly income was also significantly associated with the behavior score (*P* = .017), where it was observed that those with a monthly income of 15,000 to 24,999 SR had the highest behavior. Tukey’s post hoc test revealed no significant differences when comparing each pair. Education was also significantly associated with behavior score (*P* < .001), where it was observed that the higher the education status, the higher the behavioral score. Tukey’s post hoc test revealed that those with less than a high school education had significantly lower behavior scores than those with bachelor’s degrees (*P* < .05). Those with hereditary diseases as the cause of chronic kidney disease had a significantly higher knowledge score than those with non-hereditary disease causes (*P* = .008). Marital status, living area, best-known kidney function, and the following causes of chronic kidney disease (diabetes, cardiac disease, glomerulonephritis, renal artery stenosis, and renal stones) were not significantly associated with the knowledge score.

**Table 4:** Factors associated with behavior toward potassium-containing diet.

Factor	Behavior score		*P* value
	Mean	Standard deviation	
Age			<.001*
20–40 years	2.31	1.76	
41–60 years	1.92	1.43	
61–80 years	1.35	1.62	
Older than 80 years	0.89	1.08	
Gender			.005*
Male	2.04	1.70	
Female	1.58	1.48	
Marital status			.393
Married	1.84	1.61	
Single	1.79	1.63	
Divorced	2.38	1.82	
Occupation status			.003*
Currently employed	2.22	1.53	
Currently unemployed	1.65	1.62	
Student	2.30	1.75	
Monthly income			.017*
<4000 SR	1.75	1.67	
4000–7999 SR	1.55	1.35	
8000–14,999 SR	2.32	1.59	
15,000–24,999 SR	2.58	1.39	
>25,000 SR	2.00	2.65	
Living area			.767
Rural	1.83	1.60	
Urban	1.88	1.70	
Education status			<.001*
Less than high school	1.58	1.58	
High school	2.05	1.80	
Diploma	2.16	1.20	
Bachelor’s degree	2.55	1.64	
Master’s/PhD or equivalent	1.75	1.71	
Best known kidney function			.050
Stage 3 chronic kidney disease	1.70	1.76	
Stage 4 chronic kidney disease	1.90	1.50	
Stage 5 chronic kidney disease	2.45	1.72	
Cause of CKD			
Diabetes	1.63	1.57	.071
Hypertension	2.01	1.60	.027*
Cardiac disease	1.79	1.73	.814
Hereditary diseases	2.38	1.71	.012*
Glomerulonephritis	2.11	1.51	.297
Renal artery stenosis	2.00	1.76	.664
Renal stones	1.82	1.70	.931

CKD = chronic kidney disease.

* Significant at level .05.

## 4. Discussion

This study aimed to obtain significant insights into the knowledge and behavior of patients with CKD regarding meals with high potassium content. The results indicated that patients lacked knowledge about dietary potassium and their adherence to it was not optimal.

### 4.1. Knowledge assessment

Our study revealed that individuals with CKD exhibit a notable lack of awareness regarding diets containing potassium. Based on these data, there is a substantial need for improvement in patient education. The mean score for knowledge was 13.69 out of 52, with a standard deviation of 11.3. Additionally, a significant majority of participants (79.7%) demonstrated poor levels of knowledge. Previous studies^[17,18]^ have also shown a lack of dietary awareness among individuals with chronic kidney disease, supporting this conclusion.

The study findings revealed that 55.4% of the participants knew the potential health issues associated with excessive potassium consumption. The lack of awareness regarding the dangers associated with hyperkalemia is concerning, especially considering its potentially life-threatening consequences in patients with CKD.^[19]^ Furthermore, the high percentage of participants (80.4%) lacking awareness regarding the recommended maximum daily potassium intake for individuals with CKD underscores the pressing need for targeted nutritional education.

It is worth mentioning that individuals from various demographic groups exhibited significant variation in their levels of expertise. Younger patients (between the ages of 20 and 40), male, employed, and with higher education levels, had higher knowledge scores. Previous studies have shown that certain factors, such as sociodemographic variables, can impact health literacy and knowledge of dietary practices related to managing chronic illnesses.^[20,21]^ It is essential to tailor educational treatments to meet the specific needs of different patient groups, particularly older patients and those with lower educational attainment. Based on the identified discrepancies, it was necessary to proceed with this action.

### 4.2. Behavioral assessment

Based on the results of the behavioral evaluation, there was a noticeable difference between patients’ understanding of the importance of reducing their dietary potassium intake and the actions they ultimately took. The average behavior score was 1.84 out of 5, with a standard deviation 1.62. Interestingly, over half of the participants (52.7%) emphasized the importance of reducing potassium intake in their diet. The gap between knowledge and practice poses a significant challenge for managing chronic illnesses through dietary changes. This issue has been extensively discussed in various studies involving individuals with CKD.^[22,23]^

Various factors may have contributed to this discrepancy. Our research findings indicated that a mere 16.1% of participants considered the potassium content information on nutritional labels to be a significant factor influencing their food choices during grocery shopping. Several factors may contribute to the low proportion of people who understand the information on food labels. One factor could be the information’s complexity, making it difficult for individuals to comprehend. Additionally, many individuals may not fully grasp the importance of this information or its relevance to their dietary choices. Furthermore, it is worth noting that many product labels do not provide potassium, which further limits consumers’ ability to make informed decisions. Improving dietary adherence can be achieved by changing food labeling practices and providing patients with the necessary knowledge to utilize this information effectively.

According to the study findings, younger patients, male, and had higher levels of education exhibited better behavioral ratings. Based on the observed correlation between the knowledge scores and eating patterns, it can be concluded that more excellent knowledge is associated with better eating habits. Additional evidence of the link can be seen through the moderately positive correlation between the knowledge and conduct scores (correlation coefficient = 0.458, *P* < .001). The data shows that educational interventions specifically targeted can significantly impact and improve dietary adherence.^[24]^

### 4.3. Barriers to dietary adherence

When developing effective treatments, understanding the barriers that hinder individuals from following their diets provides valuable insight. A significant number of participants highlighted several key challenges they faced. These included the struggle to choose appropriate meals (61.1% of participants), difficulty tracking nutrient intake (54.7%), and the challenge of maintaining dietary control in social situations (over 50% of participants). Previous research has shown that individuals with CKD face challenges related to their nutrition, which aligns with the findings of this study.^[25,26]^

Approximately 45.8% of the participants expressed difficulty understanding the complex nature of the CKD diet. Patient adherence may be significantly influenced by the complexity of the treatment regimen, as well as feelings of deprivation and perceptions of the food as tasteless or bland, which reported by 43.1% of patients. The findings of this study emphasize the need to streamline dietary guidelines and explore creative approaches to enhance palatability and satisfaction with renal diets.^[27]^

Economic concerns were not a significant barrier, as only 27.2% of participants perceived the diet as costly. Several studies on different populations have indicated that cost is a substantial obstacle to adherence.^[28]^ This conclusion differs from those of other studies. Further investigation is necessary to understand the potential cultural or economic factors specific to the Jazan region that may have contributed to this discrepancy.

### 4.4. Clinical implications and future directions

This study provides valuable insights into areas that require improvement in clinical practice and will inform future research on managing CKD patients’ dietary requirements. One aspect to consider is the creation of personalized educational interventions tailored to an individual’s age and history. These interventions strongly emphasize practical abilities such as understanding food labels and managing portion sizes. A multidisciplinary approach involving psychologists, dietitians, and nephrologists is recommended to effectively address the medical and psychological aspects of dietary adherence. Developing user-friendly technical solutions, such as mobile applications, could help patients monitor their potassium intake effectively. Including patients’ family members and friends in educational programs could potentially enhance adherence to dietary restrictions, considering the challenges that arise when trying to follow these limitations in social situations. Collaborating with culinary specialists to create nutritious, low-potassium foods may help address the challenges related to diet palatability and feelings of deprivation. Ultimately, this study underscores the significance of enacting regulatory changes to improve food labeling, specifically potassium content. This will empower individuals to make informed choices regarding their dietary needs. Combining these diverse methods aims to improve patient comprehension, promote adherence to nutritional guidelines, and ultimately enhance health outcomes in individuals with CKD.

### 4.5. Limitations and future research

Further research should address the shortcomings of the present study. This study’s cross-sectional form hinders our ability to establish causal links between knowledge, conduct, and outcomes. It is necessary to conduct longitudinal research to gain a deeper understanding of the evolution of learning and behavior and their impact on clinical outcomes. Furthermore, the study was conducted in a specific region of Saudi Arabia, potentially restricting its applicability to a broader population. A comprehensive understanding of these issues can be achieved through multi-center studies encompassing diverse geographical areas and cultural contexts.

Future research should prioritize developing and evaluating treatments aimed at addressing the identified knowledge gaps and challenges to adherence. To provide valuable evidence for informing treatment, conducting randomized controlled trials that assess the efficacy of different educational strategies, technological tools, and culinary interventions would be advantageous.

## 5. Conclusion

This study provides valuable insights into the knowledge and behavior of CKD patients in Jazan, Saudi Arabia, regarding high-potassium meals. Based on these data, it is evident that there are significant disparities in patient comprehension and adherence to dietary recommendations, along with substantial obstacles to compliance. The findings emphasize the importance of improving patient education, creating personalized treatments, and adopting a multidisciplinary approach to managing nutrition in CKD. If these concerns are adequately dealt with, healthcare professionals can improve dietary adherence and, in turn, improve clinical outcomes for patients with CKD.

## Author contributions

**Conceptualization:** Atheer Akoor, Eman Refaei, Raghad Abdu Mobaraki, Naif Gharwi, Khalid Ahmed Muafa.

**Data curation:** Atheer Akoor, Mohsen Deibaji, Saleh Ghulaysi, Raghad Abdu Mobaraki.

**Formal analysis:** Mohammed Somaili, Mona Alshahrani.

**Investigation:** Eman Refaei, Naif Gharwi.

**Methodology:** Mohsen Deibaji, Abdulrahman Abdullah Aqeel, Entsar Ahmed Qadah, Abdulla Madkali.

**Resources:** Abdulrahman Abdullah Aqeel, Saleh Ghulaysi, Entsar Ahmed Qadah, Abdulla Madkali, Khalid Ahmed Muafa, Abdulrahman Hakami.

**Writing – original draft:** Areej Siddiq Areeshi, Mona Alshahrani.

**Writing – review & editing:** Mohammed Somaili, Abdulrahman Hakami, Mona Alshahrani.

## Supplementary Material


