# Supplementary material for: Cross-sectional study for assessment of knowledge, attitudes and practices of chronic kidney disease patients toward potassium-rich diet intake in Jazan-Saudi Arabia
Source: Medicine (Baltimore). 2025 May 9;104(19):e42260. doi: 10.1097/MD.0000000000042260 (PMC12074114; doi:10.1097/MD.0000000000042260)
Supplement: Supplementary file 1 [file medi-104-e42260-s001.pdf]

Appendix: Table 1

Participants Knowledge Assessment toward Potassium-Containing Diet (n = 404)

Question	n	%
Q1/ Have you been taught before about a potassium-containing diet?		
Yes (correct answer)	226	55.9
No	81	20
I don't remember	97	24
Q2/ In your opinion, do you think that too much potassium in your diet could cause a serious health problem?		
Yes (correct answer)	224	55.4
No	25	6.2
I don't know	155	38.4
Q3/ What sort of serious health problem do you think a high-potassium diet could cause? (more than one answer can be chosen)		
Muscle weakness (correct answer)	156	38.60
Numbness (correct answer)	143	35.40
Irregular heartbeats (correct answer)	136	33.70
Nausea and vomiting (correct answer)	152	37.60
Shortness of breath (correct answer)	116	28.70
Abdominal pain	106	26.20
Headache	86	21.30
Blurred vision	79	19.60
Q4/ Up to best of your knowledge, what is the maximum intake of potassium (g/day) in CKD patients?		
2 g / day (correct answer)	53	13.1
3 g / day	15	3.7
4 g / day	11	2.7
I don't know	325	80.4
Q5/ To the best of your knowledge, how much potassium do the following foods contain (answer with high or low):		
Vegetables		
Spinach (correct answer: high)		
Low	57	14.1
High	77	19.1
I don't know	270	66.8
Broccoli (correct answer: high)		
Low	61	15.1
High	54	13.4
I don't know	289	71.5
Cabbage (correct answer: low)		
Low	70	17.3
High	56	13.9
I don't know	278	68.8
Asparagus (correct answer: low)		
Low	43	10.6
High	15	3.7
I don't know	346	85.6
Cauliflower (correct answer: low)		
Low	56	13.9
High	33	8.2
I don't know	315	78
Lettuce (correct answer: low)		

Low	98	24.3
High	69	17.1
I don't know	237	58.7
Carrots (correct answer: high)		
Low	90	22.3
High	72	17.8
I don't know	242	59.9
Onions (correct answer: low)		
Low	103	25.5
High	63	15.6
I don't know	238	58.9
Beetroot (correct answer: high)		
Low	65	16.1
High	53	13.1
I don't know	286	70.8
Tomatoes (correct answer: high)		
Low	67	16.6
High	156	38.6
I don't know	181	44.8
Potato (correct answer: high)		
Low	74	18.3
High	123	30.4
I don't know	207	51.2
Cucumbers (correct answer: low)		
Low	116	28.7
High	49	12.1
I don't know	239	59.2
Pumpkins (correct answer: high)		
Low	63	15.6
High	29	7.2
I don't know	312	77.2
Eggplant (correct answer: low)		
Low	100	24.8
High	46	11.4
I don't know	258	63.9
Fruits		
Bananas (correct answer: high)		
Low	35	8.7
High	221	54.7
I don't know	148	36.6
Papayas (correct answer: high)		
Low	54	13.4
High	73	18.1
I don't know	277	68.6
Orange (correct answer: high)		
Low	75	18.6
High	130	32.2
I don't know	199	49.3

Mango (correct answer: high)		
Low	55	13.6
High	138	34.2
I don't know	211	52.2
Cantaloup (correct answer: high)		
Low	73	18.1
High	74	18.3
I don't know	257	63.6
Apple (correct answer: low)		
Low	130	32.2
High	55	13.6
I don't know	219	54.2
Apricot (correct answer: low)		
Low	69	17.1
High	64	15.8
I don't know	271	67.1
Pineapple (correct answer: low)		
Low	89	22
High	61	15.1
I don't know	254	62.9
Beaches (correct answer: low)		
Low	72	17.8
High	53	13.1
I don't know	279	69.1
Grapes (correct answer: low)		
Low	100	24.8
High	55	13.6
I don't know	249	61.6
Poultry, Diary, and Animal Products		
Chicken (correct answer: high)		
Low	70	17.3
High	119	29.5
I don't know	215	53.2
Beef, mutton (correct answer: high)		
Low	50	12.4
High	145	35.9
I don't know	209	51.7
See food (correct answer: high)		
Low	49	12.1
High	144	35.6
I don't know	211	52.2
Egg (correct answer: high)		
Low	41	10.1
High	143	35.4
I don't know	220	54.5
Milk (correct answer: high)		
Low	47	11.6
High	144	35.6

I don't know	213	52.7
Cheese (correct answer: high)		
Low	42	10.4
High	145	35.9
I don't know	217	53.7
Yogurt (correct answer: high)		
Low	58	14.4
High	131	32.4
I don't know	215	53.2
Other Types of Foods		
Beans (correct answer: high)		
Low	57	14.1
High	84	20.8
I don't know	263	65.1
Peas (correct answer: high)		
Low	66	16.3
High	84	20.8
I don't know	254	62.9
Walnuts (correct answer: high)		
Low	50	12.4
High	81	20
I don't know	273	67.6
Almonds (correct answer: high)		
Low	43	10.6
High	89	22
I don't know	272	67.3
Chickpeas/Hommos (correct answer: high)		
Low	40	9.9
High	81	20
I don't know	283	70
Lentil (correct answer: high)		
Low	47	11.6
High	101	25
I don't know	256	63.4
Chocolate (correct answer: high)		
Low	48	11.9
High	100	24.8
I don't know	256	63.4
Rice (correct answer: low)		
Low	85	21
High	79	19.6
I don't know	240	59.4
Pasta (correct answer: low)		
Low	72	17.8
High	76	18.8
I don't know	256	63.4
Noddles (correct answer: low)		
Low	66	16.3

High	71	17.6
I don't know	267	66.1
Not whole-grain bread (correct answer: low)		
Low	88	21.8
High	84	20.8
I don't know	232	57.4
Coffee cup (correct answer: low)		
Low	78	19.3
High	90	22.3
I don't know	236	58.4
Tea cup (correct answer: low)		
Low	97	24
High	69	17.1
I don't know	238	58.9
Knowledge Score (Lowest possible score = 0, highest possible score 52)		
Minimum	0	
Maximum	45	
Mean	13.69	
Standard deviation	11.3	
