# Supplementary material for: Cross-sectional study for assessment of knowledge, attitudes and practices of chronic kidney disease patients toward potassium-rich diet intake in Jazan-Saudi Arabia
Source: Medicine (Baltimore). 2025 May 9;104(19):e42260. doi: 10.1097/MD.0000000000042260 (PMC12074114; doi:10.1097/MD.0000000000042260)
Supplement: Supplementary file 2 [file medi-104-e42260-s002.pdf]

Appendix: Table 2A

Participants practices and behavior toward Potassium-Containing Diet consumption (n = 404)

**Question**	n	%
Q1/ In a typical week, on how many days do you eat the following?		
Vegetables		
Spinach		
Never or less than once per month	286	70.8
1 - 3 times per month	35	8.7
2 - 4 times per work	17	4.2
Once per day	31	7.7
2 - 3 times per day	2	0.5
I don't know	33	8.2
Broccoli		
Never or less than once per month	282	69.8
1 - 3 times per month	44	10.9
2 - 4 times per work	15	3.7
5 - 6 times per work	1	0.2
Once per day	27	6.7
2 - 3 times per day	2	0.5
I don't know	33	8.2
Cabbage		
Never or less than once per month	205	50.7
1 - 3 times per month	58	14.4
2 - 4 times per work	34	8.4
5 - 6 times per work	1	0.2
Once per day	71	17.6
2 - 3 times per day	4	1
4 - 5 times per day	1	0.2
I don't know	30	7.4
Asparagus		
Never or less than once per month	306	75.7
1 - 3 times per month	14	3.5
2 - 4 times per work	17	4.2
5 - 6 times per work	1	0.2
Once per day	23	5.7
2 - 3 times per day	2	0.5
I don't know	41	10.1
Cauliflower		
Never or less than once per month	289	71.5
1 - 3 times per month	31	7.7
2 - 4 times per work	16	4
5 - 6 times per work	4	1
Once per day	32	7.9
2 - 3 times per day	2	0.5
I don't know	30	7.4
Lettuce		
Never or less than once per month	129	31.9
1 - 3 times per month	74	18.3
2 - 4 times per work	71	17.6

5 - 6 times per work	16	4
Once per day	90	22.3
2 - 3 times per day	8	2
4 - 5 times per day	1	0.2
I don't know	15	3.7
Carrots		
Never or less than once per month	100	24.8
1 - 3 times per month	51	12.6
2 - 4 times per work	79	19.6
5 - 6 times per work	21	5.2
Once per day	123	30.4
2 - 3 times per day	17	4.2
4 - 5 times per day	1	0.2
I don't know	12	3
Onions		
Never or less than once per month	69	17.1
1 - 3 times per month	38	9.4
2 - 4 times per work	83	20.5
5 - 6 times per work	29	7.2
Once per day	143	35.4
2 - 3 times per day	24	5.9
4 - 5 times per day	5	1.2
I don't know	13	3.2
Beetroot		
Never or less than once per month	263	65.1
1 - 3 times per month	43	10.6
2 - 4 times per work	22	5.4
5 - 6 times per work	3	0.7
Once per day	39	9.7
2 - 3 times per day	5	1.2
4 - 5 times per day	2	0.5
I don't know	27	6.7
Tomatoes		
Never or less than once per month	72	17.8
1 - 3 times per month	33	8.2
2 - 4 times per work	109	27
5 - 6 times per work	32	7.9
Once per day	108	26.7
2 - 3 times per day	27	6.7
4 - 5 times per day	11	2.7
I don't know	12	3
Potato		
Never or less than once per month	78	19.3
1 - 3 times per month	44	10.9
2 - 4 times per work	93	23
5 - 6 times per work	32	7.9
Once per day	111	27.5
2 - 3 times per day	22	5.4
4 - 5 times per day	6	1.5
I don't know	18	4.5

**Cucumbers**		
Never or less than once per month	68	16.8
1 - 3 times per month	35	8.7
2 - 4 times per work	99	24.5
5 - 6 times per work	41	10.1
Once per day	127	31.4
2 - 3 times per day	22	5.4
4 - 5 times per day	2	0.5
I don't know	10	2.5
**Pumpkins**		
Never or less than once per month	290	71.8
1 - 3 times per month	39	9.7
2 - 4 times per work	15	3.7
5 - 6 times per work	2	0.5
Once per day	24	5.9
2 - 3 times per day	4	1
I don't know	30	7.4
**Eggplant**		
Never or less than once per month	117	29
1 - 3 times per month	60	14.9
2 - 4 times per work	82	20.3
5 - 6 times per work	21	5.2
Once per day	99	24.5
2 - 3 times per day	5	1.2
4 - 5 times per day	2	0.5
I don't know	18	4.5
**Fruits**		
**Bananas**		
Never or less than once per month	155	38.4
1 - 3 times per month	69	17.1
2 - 4 times per work	56	13.9
5 - 6 times per work	13	3.2
Once per day	88	21.8
2 - 3 times per day	6	1.5
I don't know	17	4.2
**Papayas**		
Never or less than once per month	246	60.9
1 - 3 times per month	66	16.3
2 - 4 times per work	22	5.4
5 - 6 times per work	5	1.2
Once per day	35	8.7
2 - 3 times per day	3	0.7
I don't know	27	6.7
**Orange**		
Never or less than once per month	148	36.6
1 - 3 times per month	68	16.8
2 - 4 times per work	72	17.8
5 - 6 times per work	11	2.7
Once per day	78	19.3
2 - 3 times per day	6	1.5

I don't know	21	5.2
Mango		
Never or less than once per month	197	48.8
1 - 3 times per month	81	20
2 - 4 times per work	40	9.9
5 - 6 times per work	5	1.2
Once per day	60	14.9
2 - 3 times per day	2	0.5
I don't know	19	4.7
Cantaloup		
Never or less than once per month	176	43.6
1 - 3 times per month	100	24.8
2 - 4 times per work	35	8.7
5 - 6 times per work	4	1
Once per day	64	15.8
2 - 3 times per day	2	0.5
I don't know	23	5.7
Apple		
Never or less than once per month	100	24.8
1 - 3 times per month	63	15.6
2 - 4 times per work	84	20.8
5 - 6 times per work	28	6.9
Once per day	100	24.8
2 - 3 times per day	10	2.5
4 - 5 times per day	2	0.5
I don't know	17	4.2
Apricot		
Never or less than once per month	231	57.2
1 - 3 times per month	71	17.6
2 - 4 times per work	19	4.7
5 - 6 times per work	3	0.7
Once per day	49	12.1
2 - 3 times per day	2	0.5
4 - 5 times per day	2	0.5
I don't know	27	6.7
Pineapple		
Never or less than once per month	193	47.8
1 - 3 times per month	88	21.8
2 - 4 times per work	29	7.2
5 - 6 times per work	6	1.5
Once per day	56	13.9
2 - 3 times per day	2	0.5
I don't know	30	7.4
Beaches		
Never or less than once per month	212	52.5
1 - 3 times per month	73	18.1
2 - 4 times per work	22	5.4
5 - 6 times per work	7	1.7
Once per day	57	14.1
2 - 3 times per day	4	1

I don't know	29	7.2
Grapes		
Never or less than once per month	130	32.2
1 - 3 times per month	77	19.1
2 - 4 times per work	62	15.3
5 - 6 times per work	13	3.2
Once per day	88	21.8
2 - 3 times per day	6	1.5
4 - 5 times per day	1	0.2
I don't know	27	6.7
Poultry, Diary, and Animal Products		
Chicken		
Never or less than once per month	120	29.7
1 - 3 times per month	33	8.2
2 - 4 times per work	91	22.5
5 - 6 times per work	28	6.9
Once per day	99	24.5
2 - 3 times per day	14	3.5
4 - 5 times per day	5	1.2
I don't know	14	3.5
Beef, mutton		
Never or less than once per month	120	29.7
1 - 3 times per month	41	10.1
2 - 4 times per work	68	16.8
5 - 6 times per work	23	5.7
Once per day	105	26
2 - 3 times per day	23	5.7
4 - 5 times per day	5	1.2
I don't know	19	4.7
See food		
Never or less than once per month	124	30.7
1 - 3 times per month	39	9.7
2 - 4 times per work	64	15.8
5 - 6 times per work	23	5.7
Once per day	114	28.2
2 - 3 times per day	15	3.7
4 - 5 times per day	4	1
I don't know	21	5.2
Egg		
Never or less than once per month	109	27
1 - 3 times per month	35	8.7
2 - 4 times per work	85	21
5 - 6 times per work	35	8.7
Once per day	101	25
2 - 3 times per day	17	4.2
4 - 5 times per day	4	1
I don't know	18	4.5
Milk		
Never or less than once per month	61	15.1
1 - 3 times per month	20	5

2 - 4 times per work	96	23.8
5 - 6 times per work	58	14.4
Once per day	133	32.9
2 - 3 times per day	15	3.7
4 - 5 times per day	4	1
I don't know	17	4.2
Cheese		
Never or less than once per month	118	29.2
1 - 3 times per month	82	20.3
2 - 4 times per work	74	18.3
5 - 6 times per work	16	4
Once per day	92	22.8
2 - 3 times per day	7	1.7
4 - 5 times per day	1	0.2
I don't know	14	3.5
Yogurt		
Never or less than once per month	101	25
1 - 3 times per month	71	17.6
2 - 4 times per work	81	20
5 - 6 times per work	21	5.2
Once per day	113	28
2 - 3 times per day	4	1
I don't know	13	3.2
Other Types of Foods		
Beans		
Never or less than once per month	199	49.3
1 - 3 times per month	57	14.1
2 - 4 times per work	33	8.2
5 - 6 times per work	10	2.5
Once per day	75	18.6
2 - 3 times per day	4	1
4 - 5 times per day	3	0.7
I don't know	23	5.7
Peas		
Never or less than once per month	157	38.9
1 - 3 times per month	68	16.8
2 - 4 times per work	53	13.1
5 - 6 times per work	10	2.5
Once per day	88	21.8
2 - 3 times per day	4	1
4 - 5 times per day	2	0.5
I don't know	22	5.4
Walnuts		
Never or less than once per month	279	69.1
1 - 3 times per month	41	10.1
2 - 4 times per work	21	5.2
5 - 6 times per work	4	1
Once per day	23	5.7
2 - 3 times per day	2	0.5
4 - 5 times per day	3	0.7

I don't know	31	7.7
**Almonds**		
Never or less than once per month	270	66.8
1 - 3 times per month	46	11.4
2 - 4 times per work	17	4.2
5 - 6 times per work	4	1
Once per day	31	7.7
2 - 3 times per day	3	0.7
4 - 5 times per day	4	1
I don't know	29	7.2
**Chickpeas/Hommos**		
Never or less than once per month	262	64.9
1 - 3 times per month	46	11.4
2 - 4 times per work	22	5.4
5 - 6 times per work	6	1.5
Once per day	34	8.4
2 - 3 times per day	7	1.7
I don't know	27	6.7
**Lentil**		
Never or less than once per month	163	40.3
1 - 3 times per month	63	15.6
2 - 4 times per work	59	14.6
5 - 6 times per work	13	3.2
Once per day	75	18.6
2 - 3 times per day	6	1.5
I don't know	25	6.2
**Chocolate**		
Never or less than once per month	219	54.2
1 - 3 times per month	42	10.4
2 - 4 times per work	42	10.4
5 - 6 times per work	15	3.7
Once per day	55	13.6
2 - 3 times per day	10	2.5
4 - 5 times per day	3	0.7
I don't know	18	4.5
**Rice**		
Never or less than once per month	44	10.9
1 - 3 times per month	24	5.9
2 - 4 times per work	70	17.3
5 - 6 times per work	64	15.8
Once per day	173	42.8
2 - 3 times per day	15	3.7
4 - 5 times per day	5	1.2
I don't know	9	2.2
**Pasta**		
Never or less than once per month	141	34.9
1 - 3 times per month	65	16.1
2 - 4 times per work	61	15.1
5 - 6 times per work	26	6.4
Once per day	89	22

2 - 3 times per day	7	1.7
I don't know	15	3.7
Noddles		
Never or less than once per month	232	57.4
1 - 3 times per month	44	10.9
2 - 4 times per work	31	7.7
5 - 6 times per work	17	4.2
Once per day	48	11.9
2 - 3 times per day	6	1.5
4 - 5 times per day	2	0.5
I don't know	24	5.9
Not whole-grain bread		
Never or less than once per month	84	20.8
1 - 3 times per month	17	4.2
2 - 4 times per work	72	17.8
5 - 6 times per work	50	12.4
Once per day	131	32.4
2 - 3 times per day	27	6.7
4 - 5 times per day	6	1.5
I don't know	17	4.2
Coffee cup		
Never or less than once per month	118	29.2
1 - 3 times per month	36	8.9
2 - 4 times per work	63	15.6
5 - 6 times per work	44	10.9
Once per day	103	25.5
2 - 3 times per day	25	6.2
4 - 5 times per day	5	1.2
I don't know	10	2.5
Tea cup		
Never or less than once per month	98	24.3
1 - 3 times per month	33	8.2
2 - 4 times per work	54	13.4
5 - 6 times per work	49	12.1
Once per day	114	28.2
2 - 3 times per day	34	8.4
4 - 5 times per day	13	3.2
I don't know	9	2.2

Appendix: Table 2B

Participants practices and behavior toward Potassium-Containing Diet consumption (n = 404)

Question	n	%
Q1/ How important to you is lowering the potassium in your diet?		
I don't know	132	32.7
Not at all important	13	3.2
Somewhat important	46	11.4
Very important	213	52.7
Q2/ Does the information about salt quantity reported on nutritional labels affect your food choices when grocery shopping?		
Never	219	54.2
Sometimes	120	29.7
Always	65	16.1
Q3/ What do you normally do to eat little potassium?		
I avoid eating, or I reduce the consumption of products rich in potassium		
No / NA	190	47.00
Yes	214	53.00
I buy alternative products, with a low potassium content		
No / NA	257	63.60
Yes	147	36.40
I read the potassium content on the nutritional labels		
No / NA	299	74
Yes	105	26
**Behavior Score (Lowest possible score = 0, highest possible score 5)**		
Minimum	0	
Maximum	5	
Mean	1.84	
Standard deviation	1.62	
